# Cleavage of Model Substrates by *Arabidopsis thaliana* PRORP1 Reveals New Insights into Its Substrate Requirements

**DOI:** 10.1371/journal.pone.0160246

**Published:** 2016-08-05

**Authors:** Guanzhong Mao, Tien-Hao Chen, Abhishek S. Srivastava, David Kosek, Pradip K. Biswas, Venkat Gopalan, Leif A. Kirsebom

**Affiliations:** 1 Department of Cell and Molecular Biology, Box 596, Biomedical Centre, SE-751 24, Uppsala, Sweden; 2 Department of Chemistry & Biochemistry, Center for RNA Biology, The Ohio State University, Columbus, Ohio, 43210, United States of America; Keio University, JAPAN

## Abstract

Two broad classes of RNase P trim the 5' leader of precursor tRNAs (pre-tRNAs): ribonucleoprotein (RNP)- and proteinaceous (PRORP)-variants. These two RNase P types, which use different scaffolds for catalysis, reflect independent evolutionary paths. While the catalytic RNA-based RNP form is present in all three domains of life, the PRORP family is restricted to eukaryotes. To obtain insights on substrate recognition by PRORPs, we examined the 5' processing ability of recombinant *Arabidopsis thaliana* PRORP1 (*At*PRORP1) using a panel of pre-tRNA^Ser^ variants and model hairpin-loop derivatives (pATSer type) that consist of the acceptor-T-stem stack and the T-/D-loop. Our data indicate the importance of the identity of N_-1_ (the residue immediately 5' to the cleavage site) and the N_-1_:N_+73_ base pair for cleavage rate and site selection of pre-tRNA^Ser^ and pATSer. The nucleobase preferences that we observed mirror the frequency of occurrence in the complete suite of organellar pre-tRNAs in eight algae/plants that we analyzed. The importance of the T-/D-loop in pre-tRNA^Ser^ for tight binding to *At*PRORP1 is indicated by the 200-fold weaker binding of pATSer compared to pre-tRNA^Ser^, while the essentiality of the T-loop for cleavage is reflected by the near-complete loss of activity when a GAAA-tetraloop replaced the T-loop in pATSer. Substituting the 2'-OH at N_-1_ with 2'-H also resulted in no detectable cleavage, hinting at the possible role of this 2'-OH in coordinating Mg^2+^ ions critical for catalysis. Collectively, our results indicate similarities but also key differences in substrate recognition by the bacterial RNase P RNP and *At*PRORP1: while both forms exploit the acceptor-T-stem stack and the elbow region in the pre-tRNA, the RNP form appears to require more recognition determinants for cleavage-site selection.

## Introduction

Most tRNA genes are transcribed as precursor RNAs (pre-tRNAs) with both the 5' and 3' ends having additional residues that need to be removed to generate functional, mature tRNAs. The ubiquitous ribonucleoprotein (RNP) ribonuclease P (RNase P) is responsible for removing the 5' leader from pre-tRNAs. In Bacteria, RNase P is composed of one RNA subunit and one protein subunit, while in Archaea and Eukarya four or more proteins associate with the sole RNA [1, 2]. Irrespective of origin, the catalytic activity resides in the RNase P RNA (RPR) as evident from its ability, even in the absence of associated protein cofactor(s), to mediate cleavage of pre-tRNA as well as various other natural (*e*.*g*., pre-4.5S RNA) and artificial (*e*.*g*., model hairpin loop) substrates [1–7].

In several eukaryotes, there also exists an RNA-free RNase P that is composed solely of proteins [8]. PRORP (proteinaceous RNase P) cleaves pre-tRNAs at the same site as the RNP variants, and is also involved in tRNA 5'-maturation. In *Arabidopsis thaliana*, three distinct PRORPs (*At*PRORP1, 2 and 3) are present, but an RPR has not been identified [9]. *At*PRORP1 is localized to the mitochondria and chloroplasts, while *At*PRORP2 and *At*PRORP3 are targeted to the nucleus [9]. Single-polypeptide PRORPs from *A*. *thaliana* nucleus/organelles have been characterized and shown to be active as individual entities, while the human mitochondrial native variant was purified as a complex with two other proteins [8, 9]. RNAi-mediated knock-down of *At*PRORP1 showed protein synthesis defects in chloroplasts and mitochondria, although only photosynthesis was defective and respiration was unaffected; interestingly, the effects on 5' processing of individual organellar tRNAs were not uniform [10]. To better understand these phenotypic effects and, more broadly, appreciate the choice of RNP- and protein-based RNase P for pre-tRNA/RNA processing, it is important to understand how the two variants recognize and process their substrates [10,11], the motivation for this study.

By examining cleavage of pre-tRNAs and model substrates, residues at and near the cleavage site have been demonstrated to influence both cleavage-site recognition and cleavage efficiency of bacterial ribonuclease P (for a review, see [12]). Specifically, the residue N_-1_, the discriminator base and the two C residues at the pre-tRNA 3' end, and the T-loop have key roles [7, 13–15]; for reviews, see [1, 16]. In contrast, we have little information about either the impact of individual substrate residues and chemical groups on cleavage or if members of the PRORP family process small model substrates.

Given the ability to chemically synthesize short RNAs (~50 nts), especially with desired chemical modifications, we previously invested considerable effort into design and validation of short hairpin model substrates for the RNP version of RNase P. We have now used this approach to investigate for the first time the effect of certain site-specific replacements (e.g., guanosine with inosine or a 2'-OH with a 2'-H) on substrate recognition and cleavage by *At*PRORP1. Our data show that recombinant *At*PRORP1 cleaves model hairpin loop substrates with at least a 1000-fold lower single-turnover rate than that observed for cleavage of the parental pre-tRNA (pSu1, the *Escherichia coli* tRNA^Ser^Su1 precursor). We also found a dramatic decrease in the cleavage rate upon replacement of either the 2'-hydroxyl at -1 or the seven-bp T-loop equivalent with a GAAA-tetraloop in the model substrate. Moreover, like the bacterial RPR, the -1 identity is an important cleavage-site determinant in the context of both pre-tRNA and model substrates, irrespective of whether the -1 residue is paired or not with the residue at the discriminator position. These results led to some predictions in terms of disfavored sequences for processing by *At*PRORP1. We gained support for these predictions by examining the sequences of all mitochondrial and chloroplast tRNA genes from eight different green algae and plants, an analysis not reported before. Together, these findings provide new insights into *At*PRORP1-mediated catalysis and offer possibilities to dissect the role of individual residues and chemical groups important for cleavage. We have also integrated our findings with two very recent studies on PRORP-mediated substrate recognition [17, 18] that appeared during preparation of this manuscript.

## Materials and Methods

### Preparation of substrates

The *Escherichia coli* tRNA^Ser^Su1 precursor (*Eco* pSu1) and its variants were generated as run-off transcripts using T7 DNA-dependent RNA polymerase and PCR-amplified templates as described elsewhere [19, 20; Mao & Kirsebom, unpublished]. The different model hairpin loop substrates, pATSer, were purchased from Dharmacon, USA, purified on a 15% (w/v) polyacrylamide/ 7M urea gel culminating in an overnight Bio-Trap extraction (Schleicher and Schuell, BmbH, Germany; Elutrap in USA and Canada). The different substrates were 5'-end-labeled with γ-[^32^P]-ATP using 30 units of T4 polynucleotide kinase (ThermoFisher Scientific) and gel-purified using standard protocols [7, 21, 22]. *Eco* RNase P RNA (*Eco* RPR) was generated as described elsewhere [23, 24].

### Preparation of substrates for binding studies

The DNA template for *in vitro* transcription of pSu1 with a 5-nt trailer was generated by PCR using primers FWD (5'-taatacgactcactata***g***atctgaatggagag-3'; the italicized *g* was added to facilitate transcription) and REV (5'-*ggtgt*cggagagagggggattt-3'; the trailer sequence added is italicized). The DNA template was the plasmid pUC19-pSu1 [20].

**Fig 1:**
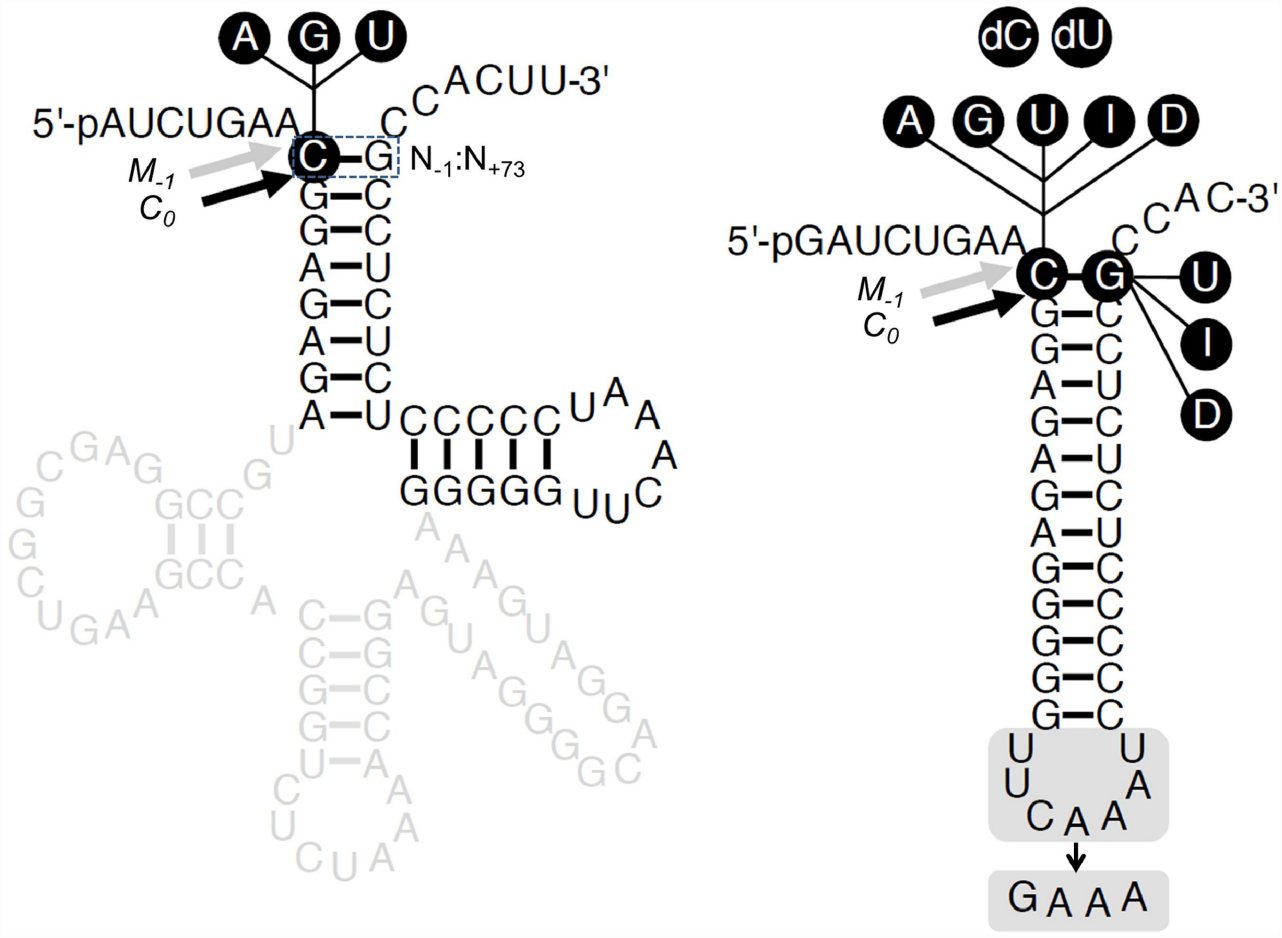
Secondary structures of substrates used in this study. Secondary structures of pSu1 and pATSer. The highlighted regions/residues were substituted to generate the different variants as indicated, A, adenosine, G, guanosine, U, uridine, I (Ino), inosine and D (DAP), 2,6-diaminopurine; dC, deoxycytosine; and dU, deoxyuridine. The canonical RNase P cleavage sites between residues N_-1_ and N_+1_ (correct cleavage denoted *C*_*0*_), and the alternative cleavage sites between residues N_-2_ and N_-1_ (miscleavage denoted as *M*_*-1*_) are marked with black and grey arrows, respectively. The N_+73_ position, which immediately precedes the 3'-terminal CCA-motif, corresponds to the discriminator base.

The DNA template for *in vitro* transcription of pATSerUG derivatives (Fig 1) were generated in two phases. In the first step, fill-in reactions were performed with two oligos: pATSerUG (5'-actcactata*gatctgaatggagagagggg*-3' and 5'-gggatttgaac*cccctctctccattcagatc*-3') and pATSerUG_GAAA_ (5'-actcactata*gatctgaatggagagagggg*-3' and 5'-gggtttc*ccccctctctccattcagatc*-3'); the overlapping regions in each pair are italicized. In the second step, the fill-in products were subjected to PCR amplification to obtain the complete sequence (including the T7 RNA polymerase promoter): for pATSerUG, the forward and reverse primers were 5'-taatacgactcactatagatctgaatg-3' and 5'-ggtgtcggagagagggggatttgaacccc-3', respectively; for pATSerUG_GAAA_, only the reverse primer was changed (5'-ggtgtcggagagagggggtttccccc-3'). The amplicons were purified and used in *in vitro* transcription as described elsewhere [25].

3'-Labeling of pSu1 and the pATSer derivatives was performed with some modifications of a previously described procedure [26–28]. For each substrate, 130 μM of *in vitro* transcribed RNA in 100 μL 100 mM NaOAc (pH 4.5) was oxidized by addition of 10 mM NaIO_4_, and incubated at 22°C for 1.5 h in the dark. The RNAs were then ethanol precipitated and re-suspended in 500 μL of 100 mM NaOAc (pH 5.2) using a 20:1 molar ratio of fluorescein-5-thiosemicarbazide (FTSC):RNA; FTSC was a generous gift of Prof. Edward Behrman, Ohio State University (OSU). The labeling reactions were carried out at 4°C for 16 h in the dark. Excess, unincorporated FTSC was removed by sequential phenol-chloroform and charcoal extractions, followed by purification using a 8% (w/v) polyacrylamide/7 M urea gel. The excised RNA was eluted at 4°C for 16 h into 1 M NaOAc (pH 4.9), and then subjected to ethanol precipitation. The 3'-labeling efficiency was typically >90%, as assessed by Abs_260_ (RNA) and Abs_492_ (fluorescein) values.

### Cleavage assays and determination of *k*_*app*_

The cleavage reactions with *At*PRORP1 (purified as described in ref. 28) were performed in buffer containing 20 mM HEPES-KOH (pH 7.4), 100 mM NH_4_OAc, 4 mM DTT, 10 mM Mg(OAc)_2_ and 0.8 mM spermidine. To determine the optimal Mg^2+^ concentration for cleavage, Mg(OAc)_2_ was added separately to give the final concentration as indicated. All assays were performed at 37°C. The reactions were terminated by adding twice the assay volume of stop solution (10 M urea, 100 mM EDTA), and the products were separated on 25% (w/v) polyacrylamide/7 M urea gels.

The rate constant k_app_ was determined under single-turnover conditions at pH 7.4 in the presence of 10 mM Mg^2+^, which was determined to be optimal for *At*PRORP1-mediated cleavage of pSu1 and pATSerUG. The concentration of *At*PRORP1 used was 0.37 μM for assays with pSu1 [except 1.1 μM for pSu1(-1C)] and 5.6 μM for assays with pATSer derivatives (except 4 μM for pATSer 3' truncated variants). The concentrations of *At*PRORP1 used to generate the data are specified in the respective figure legends. The concentration of pSu1 and model substrates was 0.02 μM. For rate calculations, we used the 5' cleavage fragment as a measure of product formed. In each assay, the time of incubation was adjusted to ensure that the velocity measurements were in the linear range (typically ≤10% but never exceeding 40%). Each k_app_ value is reported as a mean ± standard deviation of this value, which were calculated using data (six time points) from at least three independent experiments.

### Fluorescence polarization binding assays and determination of *K*_*D*_ values

Defined amounts of *At*PRORP1, as indicated, were incubated individually with either 2 nM pSu1 or 20 nM pATSer derivatives that had been 3'-labeled with fluorescein [28]. The binding reactions were performed in 20 mM HEPES (pH 7.2), 10 mM Ca(OAc)_2_, 100 mM NH_4_OAc, 4 mM DTT, and 5% (v/v) glycerol. The reactions were carried out for at least 10 min at 22–25°C in a 384-well plate (Corning Costar black round bottom). The fluorescence polarization values were then obtained using infinite M1000 PRO (Tecan), with the G factor set to 1.2. Polarization (P) observed in the presence of different *At*PRORP1 concentrations were subtracted from that observed with the respective substrate alone to obtain ΔP at each protein concentration tested. The dissociation constants were then calculated by fitting to $\Delta \text{P}=\;\frac{{\Delta \text{P}}_{\text{max}}\text{ x }\left[At\text{PRORP}1\right]}{{\text{K}}_{\text{D}}\;+\;\left[At\text{PRORP}1\right]}$ using KaleidaGraph (Synergy). The curve-fit errors for each measurement did not exceed 26%, with R^2^ values ≥ 0.96. Each K_D_ value is reported as a mean ± standard deviation, which were calculated using data from at least three independent experiments.

## Results

### The identity of N_-1_ in pre-tRNA^Ser^ (pSu1) influences cleavage by At*PRORP1*

Studying the recognition and cleavage of a suite of model substrates (pATSer series) derived from *Eco* pre-tRNA^Ser^Su1 (pSu1) by the bacterial RNase P RNP has been gainful [6, 7, 14, 15, 20–22, 29–35]. To facilitate a direct comparison of substrate recognition by the RNP and proteinaceous forms of RNase P, we therefore chose to exploit the same pAT series of model substrates. Moreover, compared to other pre-tRNAs used to study PRORP-mediated cleavage [17, 18, 36], *Eco* tRNA^Ser^ is equipped with a longer variable loop thus enabling a comparison of structurally distinct pre-tRNAs. Towards this overall objective, we first investigated if a recombinant *At*PRORP1 could cleave pSu1 [Fig 1; wild type pSu1 referred hereafter as pSu1(-1C)].

**Fig 2:**
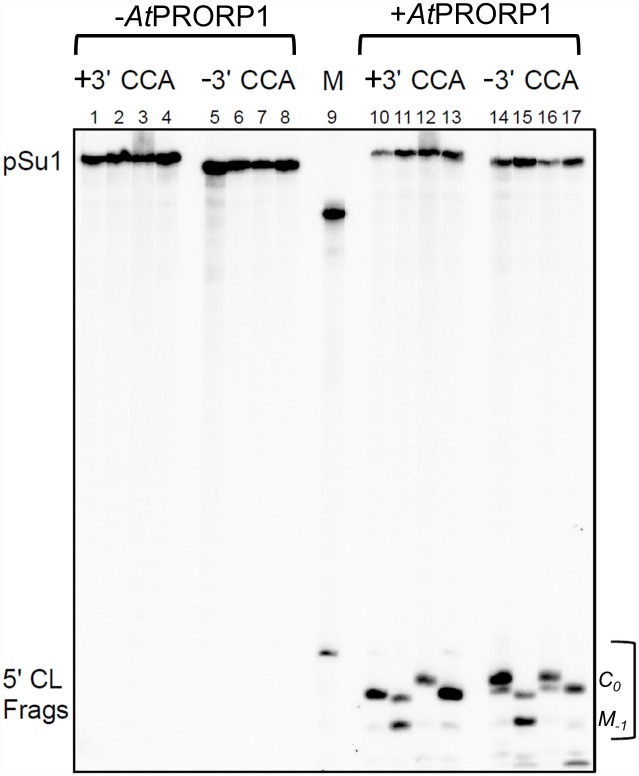
*At*PRORP1-mediated cleavage of pre-tRNA^Ser^Su1 (pSu1). Representative gel showing *At*PRORP1-mediated cleavage of pre-tRNA^Ser^Su1 (pSu1) substrates with and without the 3' CCA. Lanes 1 to 8 represent negative controls (absence of *At*PRORP1), and M (size marker, lane 9) indicates cleavage of pATSerUG by *Eco* RPR. Note that this cleavage generates a 5' cleavage fragment (5' CL Frags) one nucleotide longer compared to that generated during cleavage of pSu1. Lanes 10 and 14 pSu1(-1A), lanes 11 and 15 pSu1(-1C), lanes 12 and 16 pSu1(-1G), and lanes 13 and 17 pSu1(-1U). The final concentration of *At*PRORP1 was 0.37 μM and the reactions were performed at 37°C for 30 s in the presence of 10 mM Mg^2+^ (see Materials and Methods).

**Fig 3:**
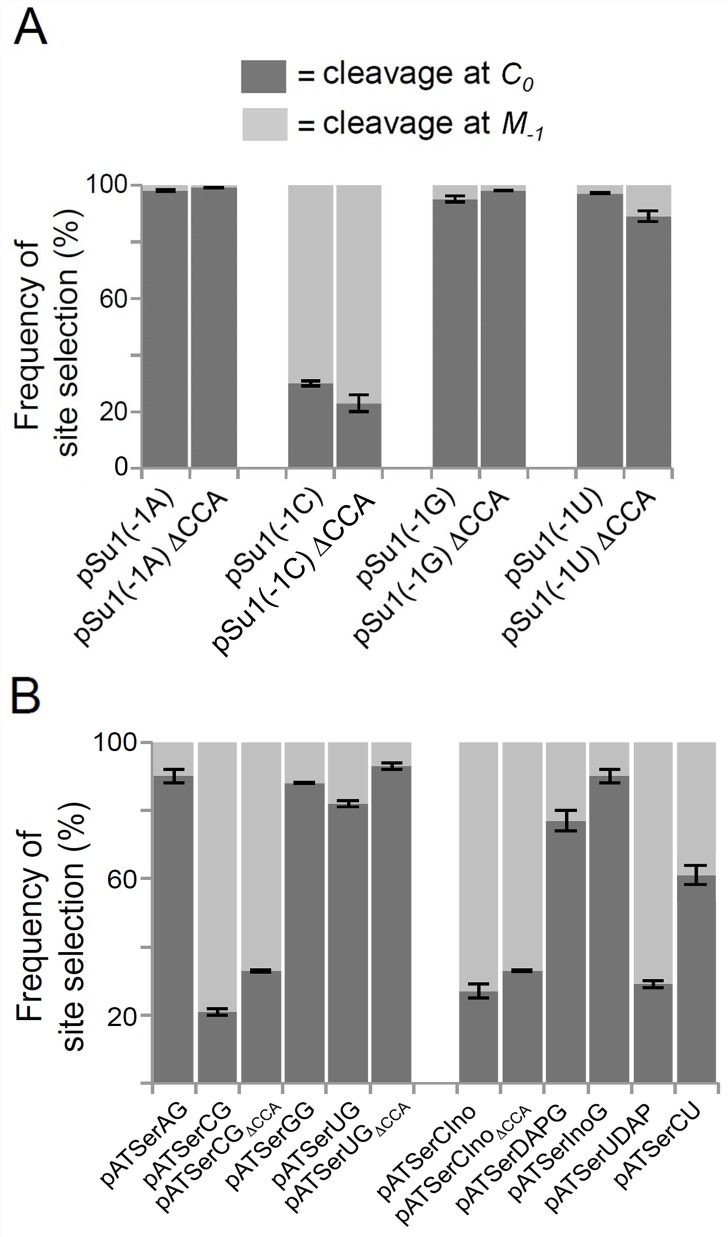
Frequencies of cleavage-site selection by *At*PRORP1. Histograms summarizing cleavage-site selection frequencies (in %) during *At*PRORP1-mediated cleavage of pSu1 "-1" (A) and pATSer (B) variants. Mean and standard deviation values were calculated using data from at least three independent experiments.

*Eco* RPR cleaves pSu1(-1C) predominantly at the canonical correct position between N_-1_ and N_+1_ (termed *C*_*0*_), but also miscleaves between N_-2_ and N_-1_ (termed *M*_*-1*_; Fig 1) [13]. In contrast, *At*PRORP1 cleaved pSu1(-1C) mainly at *M*_*-1*_ but also at *C*_*0*_ (Fig 2, lane 11; Fig 3A). Interestingly, substitution of C_-1_ with U_-1_ or A_-1_ or G_-1_ resulted in preferential cleavage at *C*_*0*_ (Fig 2). Together, these findings suggest that the identity of N_-1_ and/or pairing between N_-1_ and the discriminator base (as in C_-1_:G_73_) play an important role in cleavage-site selection.

**Fig 4:**
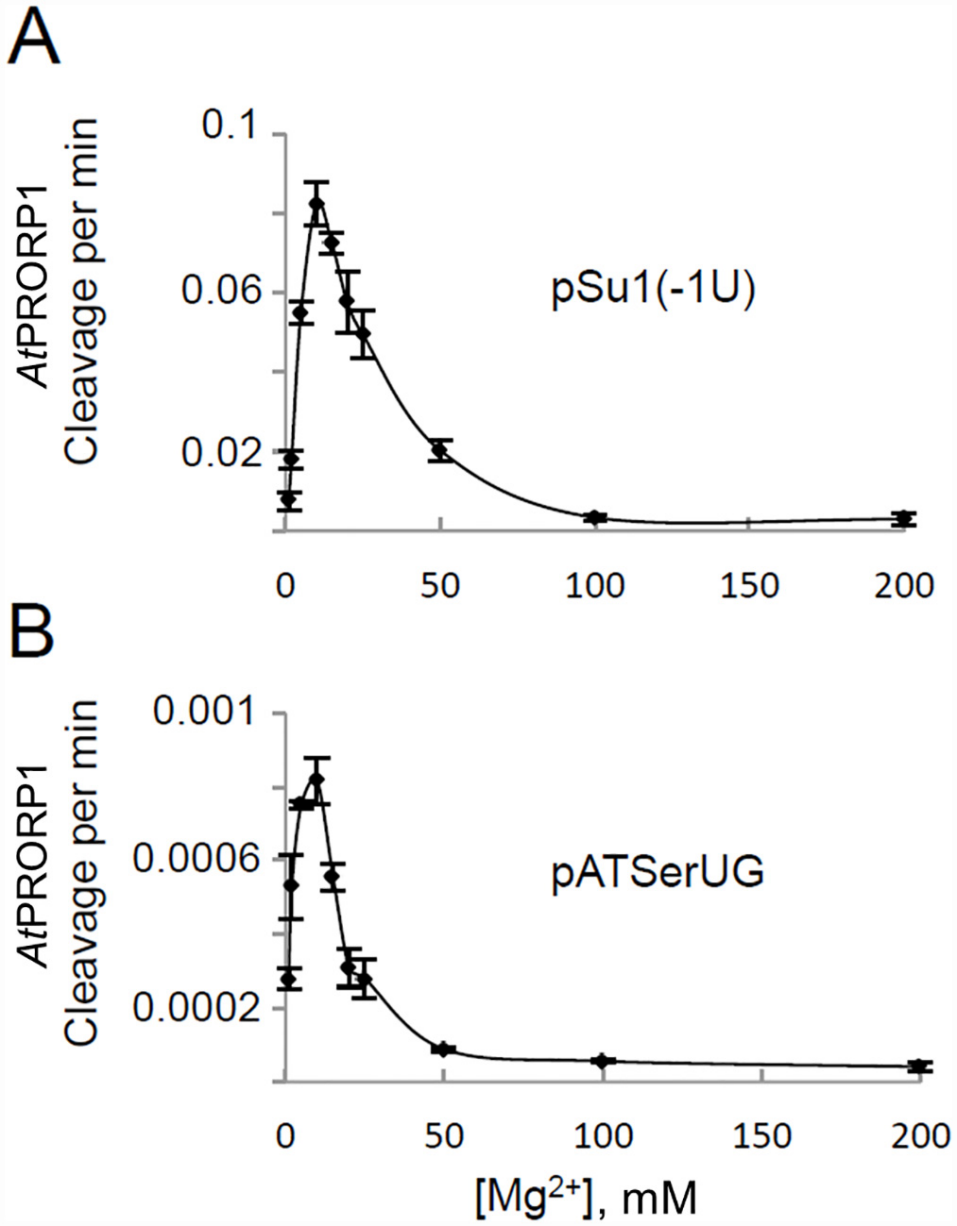
Effect of varying Mg^2+^ concentration on *At*PRORP1-mediated cleavage. *At*PRORP1-mediated cleavage of the pSu1(-1U) (A) and pATSerUG (B) as a function of Mg^2+^ concentration at 37°C. Mean and standard deviation values were calculated using data from at least three independent experiments.

Because an examination of the single- and multiple-turnover rates indicated that cleavage (or a preceding step) is likely to be rate limiting for *At*PRORP1 [37], we determined the apparent rates (k_app_) of cleavage for the pSu1 "-1 variants" under single-turnover conditions. We first determined that the optimal Mg^2+^ concentration for cleavage of pSu1(-1U) by *At*PRORP1 was 10 mM Mg^2+^ (Fig 4A); we found that the choice of cleavage site did not change with increasing Mg^2+^. Hence, we chose 10 mM Mg^2+^ for the kinetic studies.

When we examined the different model substrates for cleavage at *C*_*0*_ and *M*_*-1*_, k_app_ showed a three-fold variation with pSu1(-1C) being the weakest substrate. In contrast, k_app_ for cleavage at *M*_*-1*_ (the incorrect site) was roughly 20-fold higher for pSu1(-1C) compared to the other three N_-1_ variants (Table 1) consistent with its miscleavage propensity. Irrespective of the substrate tested, the frequency of cleavage at *M*_*-1*_ and *C*_*0*_ did not change as a function of time (not shown).

**Table 1:** Rate of cleavage (k_app_) of pSu1 and pATSer variants at 10 mM Mg^2+^.

Substrate	Cleavage site	k_app_ (min^-1^) With 3'-CCA	k_app_ (min^-1^) Without 3'-CCA
pSu1(-1C)	*C*_*0*_	0.5±0.01	0.25±0.005
	*M*_*-1*_	0.7±0.01	0.5±0.01
pSu1(-1A)	*C*_*0*_	1.6±0.01	2.5±0.1
	*M*_*-1*_	0.04±0.0004	0.02±0.001
pSu1(-1G)	*C*_*0*_	0.8±0.004	2±0.07
	*M*_*-1*_	0.03±0.001	0.02±0.0004
pSu1(-1U)	*C*_*0*_	1.4±0.08	0.4±0.01
	*M*_*-1*_	0.03±0.003	0.03±0.003
pATSerCG^#^	*C*_*0*_	0.0002±0.00003	0.0012±0.00001
	*M*_*-1*_	0.0008±0.00005	0.0022±0.0001
pATSerUG^#^	*C*_*0*_	0.0013±0.00005	0.0034±0.0002
	*M*_*-1*_	0.0002±0.000005	0.00035±0.00005
pATSerCIno^#^	*C*_*0*_	0.0004±0.000001	0.001±0.00005
	*M*_*-1*_	0.0012±0.00001	0.0019±0.00002
pATSerCU^#^	*C*_*0*_	0.002±0.00005	ND
	*M*_*-1*_	0.0014±0.00002	ND
pATSerUG^##^	*C*_*0*_	0.0009±0.0001	NA
pATSerUG_Δ3'AC_^##^	*C*_*0*_	0.0006±0.00004	NA
pATSerUG_Δ3'CAC_^##^	*C*_*0*_	0.001±0.0001	NA
pATSerUG_Δ3'CCAC_^##^	*C*_*0*_	0.003±0.0008	NA

Each value listed is a mean ± standard deviation determined from three or more independent experiments.

^#^C and U correspond to residue identity at the -1 position while G, Ino (inosine) and U refer to residue identity at the discriminator position "+73" (numbering same as in tRNA; Fig 1).

^##^k_app_ values determined at 25 mM Mg^2+^ for these substrates. While these experiments were performed prior to our establishing 10 mM Mg^2+^ as being optimal, the rate and fidelity of cleavage is largely unchanged between 10 to 25 mM Mg^2+^. Δ3'AC, Δ3'CAC and Δ3'CCAC indicates residues in the 3'CCAC motif that were deleted. ND, not determined; NA, not applicable.

#### The 3'-CCA in pre-tRNA^Ser^ (pSu1) is not a major determinant for cleavage by AtPRORP1

*Eco* pSu1 has a 3' terminal CCA-motif (Fig 1). However, eukaryotic and organellar tRNA genes in general do not encode CCA (see *e*.*g*. http://trna.ie.niigata-u.ac.jp/cgi-bin/trnadb/index.cgi.). When we analyzed the organellar tRNA sequences for 8 algal and plant species (available at http://plantrna.ibmp.cnrs.fr.), only 0.5% (2 out of 423) tRNA-encoding genes have a 3'-CCA: a choloroplast tRNA^Ala^ in *Cyanophora paradoxa* and a mitochondrial tRNA^Ile^ in *Solanum tuberosum* (potato). Thus, *At*PRORP1–localized to the mitochondria and chloroplasts—may not encounter pre-tRNAs with 3'-CCA.

Although the inference was drawn from a single end-point measurement, it was previously reported that the presence of the 3'-CCA in pre-tRNA decreases *At*PRORP1 cleavage and might therefore serve as an anti-determinant [9]. We therefore generated truncated pSu1 "-1 variants", which lack this CCA-motif (Fig 1), and assessed their fidelity and rate of cleavage by *At*PRORP1 (Fig 2, lanes 14 to 17; Fig 3A and Table 1). With respect to cleavage site-selection, we did not observe any major difference with and without the 3'-CCA, if anything a small increase in cleavage at *M*_*-1*_ for pSu1(-1C) and pSu1(-1U) in the absence 3'-CCA (Fig 3A). Upon deletion of the 3'-CCA motif, we noted a modest increase in k_app_ (at *C*_*0*_) for substrates having A_-1_ or G_-1_, while a decrease was detected for those with C_-1_ or U_-1_. The most striking effect was a 3.5-fold decrease in k_app_ for cleavage of pSu1(-1U) at *C*_*0*_ (Table 1). A simple classification that the 3'-CCA motif acts as a positive or negative determinant is not possible given the substrate-context effects.

### Cleavage of model hairpin loop substrates by At*PRORP1*

**Fig 5:**
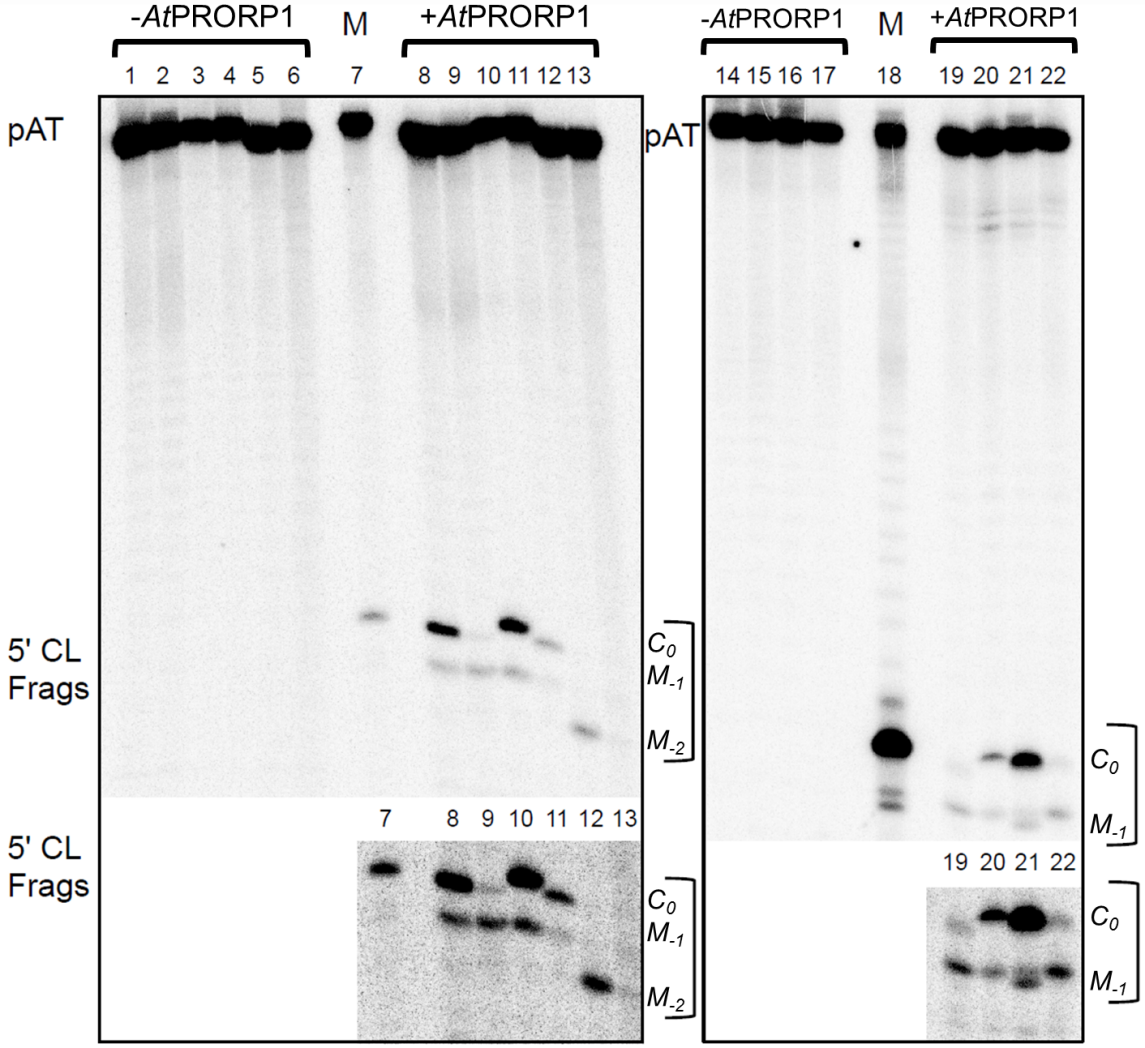
*At*PRORP1-mediated cleavage of pATSer variants. Representative gel showing *At*PRORP1-mediated cleavage of 3' CCA-motif-containing pATSer variants. Lanes 1 to 6 and 14 to 17 are negative controls (loaded in the same order as the reactions with *At*PRORP1 in lanes 8 to 13 and 19 to 22, respectively); and lanes 7 and 18 (size marker) refers to cleavage of pATSerUG by *Eco* RPR. The final concentration of *At*PRORP1 was 6.6 μM and the reactions were performed at 37°C for 60 min in the presence of 10 mM Mg^2+^. The position of each 5' cleavage fragment (5' CL Frags) generated after cleavage is indicated. The two lower panels represent overexposure to better highlight the 5'-cleavage products in the upper panels. (Note: Fig A in S1 File shows cleavage of pATSer derivatives without the 3' CCA-motif.)

We next investigated whether *At*PRORP1 cleaves the model hairpin loop substrate pATSerCG, which is composed of the 5' leader, the amino acid acceptor-stem (with the 3'CCA-motif and a dangling 3'C), and the T-stem and loop of pSu1(-1C) (Fig 1). Indeed, pATSerCG acts as a substrate for *At*PRORP1 (Fig 5, lane 9), and as expected based on the fidelity of processing of pSu1(-1C), pATSerCG was also cleaved mainly at *M*_*-1*_ (Fig 3B). Substitution of C at -1 with U (pATSerUG with and without the 3'-CCA-motif) shifted the major cleavage site to *C*_*0*_, again reminiscent of pSu1(-1U) (Fig 5; see also Fig 3B and Fig A in S1 File). Clearly, at least the determinants for cleavage-site selection are all preserved in the simpler model substrate. In fact, even the optimal [Mg^2+^] of 10 mM that we determined for cleavage of pATSerUG parallels that for pSu1(-1U) (Fig 4B).

The rates of cleavage (k_app_) of pATSerCG and pATSerUG at 10 mM Mg^2+^ were dramatically lower than their pSu1 counterparts (Table 1). For pATSerCG, the *C*_*0*_ and *M*_*-1*_ rates are 2500- and almost 900-fold lower, respectively, while for pATSerUG cleavage at *C*_*0*_ was three orders of magnitude lower. Deleting the 3'-CCA-motif resulted in a modest increase in k_app_ for both pATSerCG and pATSerUG. In this context, note that deletion of both C's and the 3' terminal A is needed to elicit a modest increase in k_app_ (Table 1).

Despite weak cleavage of the model substrates, compared to the parental pre-tRNA, the qualitative trends with respect to cleavage-site selection are similar for pSu1 and the pATSer N_-1_ variants (Fig 3). For example, comparison of pSu1(-1C) and pATSerCG (both without 3'-CCA) reveals that the k_app_ for cleavage at *M*_*-1*_ relative to *C*_*0*_ is two-fold greater in each case (Table 1). For the same cohort with 3'-CCA, the k_app_ for cleavage at *M*_*-1*_ relative to *C*_*0*_ is 1.4-fold higher for pSu1(-1C) and four-fold for pATSerCG (Table 1).

### Hairpin loop substrate binds with lower affinity than pre-tRNAs to At*PRORP1*

We next used a previously described fluorescence polarization assay [38] to determine the dissociation constants (K_D_ values) for the binding of 3'-CCA-containing pATSerUG and pSu1(-1U) to *At*PRORP1. These binding reactions were performed in the presence of Ca^2+^, because *At*PRORP1 shows tight pre-tRNA binding but no detectable cleavage when Mg^2+^ is substituted with Ca^2+^ [28, 38]. The K_D_ value for pATSerUG increased by almost 200-fold relative to pSu1(-1U) (Table 2; see also Fig B in S1 File). This change, which corresponds to a loss of 3.2 kcal/mol in binding, reflects the importance of the D stem-loop, and perhaps the T-/D-loop tertiary contacts, for tight substrate binding by *At*PRORP1. The model substrate also lacks the anticodon stem-loop, but this structural element has been shown to be dispensable for substrate recognition and cleavage by the RNP and *At*PRORP forms of RNase P [4, 6, 9, 13, 20, 39].

**Table 2:** Binding constants (K_D_) for pSu1(-1U), pATSerUG and pATSerUG_GAAA_.

Substrate	K_D_, μM	ΔΔG, kcal/mol
pSu1(-1U)	0.0063±0.0026	1
pATSerUG	1.2±0.067	-3.2
pATSerUG_GAAA_	0.93±0.18	-3.1

K_D_ values were determined at 10 mM Ca^2+^ and 25°C. Each K_D_ value is an average of at least three independent experiments. ΔΔG values were calculated using the equation ΔΔG = -RTln [K_D_ (pATSerUG or pATSerUG_GAAA_)/K_D_(pSu1(-1U)] [40].

#### C_-1_ and N_-1_:N_+73_ pairing influence cleavage by AtPRORP1

Our results show that N_-1_ identity influences cleavage by *At*PRORP1, as is particularly evident from results obtained with C_-1_ substrates that were cleaved preferentially at the alternative site *M*_*-1*_ (miscleavage). Both pSu1 and pATSer have G_+73_ as the discriminator base, and therefore have the possibility of C_-1_:G_+73_ pairing. Thus, the bp just upstream of the correct cleavage site could affect fidelity and rate. To investigate this possibility, we next generated pATSer variants with different N_-1_:N_+73_ options (Fig 1). These substrates are referred to as pATSerAG, pATSerGG, pATSerCIno (inosine at +73 can potentially form two H-bonds with C_-1_), pATSerDAPG (2,6-diamino purine at -1), pATSerInoG (inosine at -1), pATSerUDAP (2,6-diamino purine at +73 can potentially form three H-bonds with U_-1_) and pATSerCU.

The cleavage data (Figs 3 and 5) showed that pATSer acts as an *At*PRORP1 substrate irrespective of the identity of residue -1. While substrates having A_-1_, G_-1_, DAP_-1_, Ino_-1_ and U_-1_ (except for pATSerUDAP) were cleaved preferentially at the correct site, C_-1_ resulted in cleavage at *M*_*-1*_ even when it is not engaged in pairing with N_+73_ as evident from cleavage of pATSerCU (Fig 3B). Moreover, formation of a N_-1_:N_+73_ pair with three H-bonds resulted in cleavage mainly at the alternative site *M*_*-1*_ (see pATSerCG and pATSerUDAP). Together, these data suggest that C_-1_ as well as the presence of a N_-1_:N_+73_ pair with three H-bonds in a pATSer context influence the choice of cleavage site by *At*PRORP1. Consistent with these findings, the k_app_ for pATSerCU (absence of the -1/+73 pair) was ten-fold higher for cleavage at the correct site compared to pATSerCG while it was two-fold higher for pATSerCIno (Table 1).

**Fig 6:**
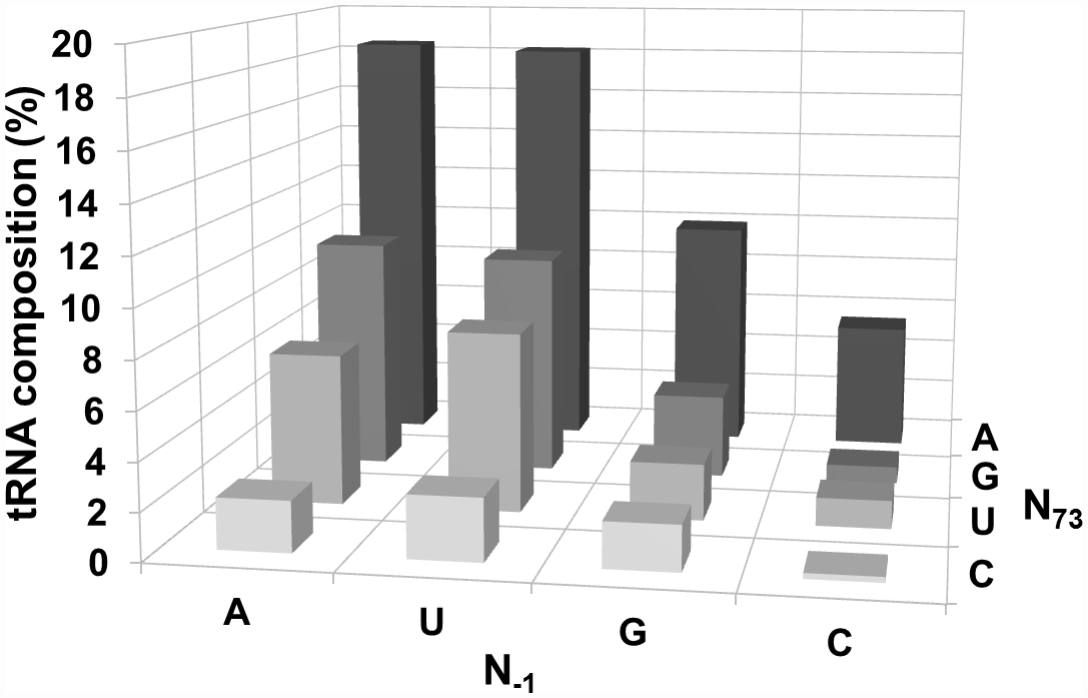
Analysis of N_-1_:N_+73_ identities in mitochondrial and chloroplast tRNAs. Analysis of N_-1_:N_+73_ identities in 423 mitochondrial and chloroplast tRNAs from eight different green algae and plants (sequences obtained from http://plantrna.ibmp.cnrs.fr/). Table A in S1 file lists the individual distributions in each species.

Since our findings indicated that N_-1_ identity and the strength of the N_-1_:N_+73_ pair play important roles in determining the rate and fidelity of cleavage, and that some combinations result in adverse effects with respect to *At*PRORP1 catalysis, we postulated that a bias might become apparent from an analysis of the N_-1_:N_+73_ sequence information among the organellar tRNAs in eight different green algae and plants (Fig 6; Table A in S1 File). From examining these 423 tRNAs, we observed the following features. First, C_-1_:G_+73_ was present in only 0.7% of the tRNAs (3 instances) even though C_-1_ displayed a 10-fold higher incidence (7.6%, 32 out of 423). Second, there were eight examples of G_-1_:C_+73_ (~2%), but seven of these were tRNA^His^; a universal identity determinant of tRNA^His^ for histidyl-tRNA synthetase is the presence of G_-1_ and an 8-bp acceptor stem. Last, there is a variable distribution of other pairing possibilities: A_-1_:U_+73_ (6.4%), U_-1_:A_+73_ (18.2%) and G_-1_:U_73_ (2.4%). Although an in-depth analysis is needed to draw firm conclusions, it appears that two hydrogen bonds in the N_-1_:N_+73_ pair alone might not engender miscleavage, especially when N_-1_ is not a C. A better understanding of cleavage-site selection as well as the biological specificity of PRORP *in vivo* requires determining the k_cat_/K_m_ for cleavage (at *C*_*0*_ and *M*_*-1*_) of different pre-tRNAs that exemplify the natural variations.

### Role of the 2'-OH at N_-1_ in cleavage by At*PRORP1*

The 2'-OH at N_-1_ in pATSer plays an important role for *Eco* RPR-mediated cleavage at the correct site [32, 33]. For comparison, we therefore decided to study *At*PRORP1 cleavage of pATSer variants in which the 2'-OH at N_-1_ was replaced with 2'-H (deoxy). These variants, pATSerU_deoxy_G and pATSerC_deoxy_G (Fig 1), were subjected to cleavage by *At*PRORP1. For the variants carrying 2'-H at N_-1_, we did not detect any cleavage at any position (Fig C in S1 File). This result is in contrast to what has been reported for *Eco* RPR-mediated cleavage of the same substrates (see Discussion).

### Role of the pATSer loop in cleavage by At*PRORP1*

Since the structure of the pATSerCG loop (corresponding to the T-loop in pSu1; Fig 1) influences cleavage efficiency and cleavage site-selection in *Eco* RPR-mediated cleavage [21, 22], we tested the significance of the loop for *At*PRORP1 catalysis. Indeed, replacing the loop in both pATSerCG and pATSerUG with a GAAA-tetraloop reduced cleavage efficiency dramatically (Fig 5 lanes 12 and 13). We detected cleavage at *M*_*-2*_ (between residues -1 and -2) and no cleavage at either *C*_*0*_ or *M*_*-1*_ for pATSerCG_GAAA_, and very little cleavage at *M*_*-2*_ (if any) or any other position for pATSerUG_GAAA_. For both these substrates we were unable to determine k_app_. Interestingly, comparing the K_D_ values for pATSerUG and pATSerUG_GAAA_ revealed that *At*PRORP1 binds both these substrates with roughly equal affinity (Table 2). Collectively, these data suggested that the structure of the loop in pATSer influences cleavage efficiency and site recognition but not binding.

## Discussion

### Requirements for efficient and accurate cleavage by At*PRORP1*

In addition to pre-tRNAs, PRORPs from various sources are capable of processing mRNAs, tRNA-like molecules called t-elements, and snoRNAs [9, 41, 42]. Here, we investigated processing of mutant derivatives of pre-tRNA^Ser^(Su1)-based substrates by *At*PRORP1 and interpret here the experimentally observed nucleobase preferences with identity biases in the sequences of organellar tRNAs. We have also drawn collectively from two recent complementary reports: Brillante *et al*. used *At*PRORP3 and *Thermus thermophilus* (*Tth*) pre-tRNA^Gly^, and Howard *et al*. compared *At*PRORP1, *At*PRORP2 and *At*PRORP3 for their ability to process both nuclear and organellar pre-tRNAs (including *Arabidopsis* mitochondrial pre-tRNA^Cys^) [17, 18]. Overall, these results are expected to contribute to an understanding of the versatility of PRORPs, comparison of substrate-recognition by PRORP and RNP-based RNase P, and possibly the driving force for evolution of the two forms.

First, we showed that *At*PRORP1 cleaves *Eco* pre-tRNA^Ser^(pSu1) that has a large variable loop, a structural feature that is known to affect the structural topography in the vicinity of the T-/D-loop region [43]. We investigated pSu1 since previous studies with PRORP variants used pre-tRNAs with smaller variable loops (*e*.*g*., pre-tRNA^Tyr^, pre-tRNA^Phe^, pre-tRNA^Gly^, pre-tRNA^Cys^ [8, 9, 17, 18, 28, 29].

Second, we demonstrate here that *At*PRORP1 cleaves model hairpin-loop substrates (45-nt pATSer variants) at least 1000-fold slower than the parental pre-tRNA^Ser^ (Table 1). Our finding is consistent with the >1000-fold decrease reported for *At*PRORP1-mediated processing of an *Arabidopsis* mitochondrial pre-tRNA^Cys^-derived stem-loop substrate compared to pre-tRNA^Cys^ [18]; this decrease was less pronounced with *At*PRORP2 and *At*PRORP3 (30- and 67-fold, respectively). Brillante et al. independently reported a 26-fold lower rate for *At*PRORP3-mediated cleavage of a *Tth* pre-tRNA^Gly^-derived stem-loop substrate relative to pre-tRNA^Gly^ [17]. Given the striking similarity of the tertiary structures of *At*PRORP1 and *At*PRORP2 [38, 44], their differences in processing stem-loop substrates is surprising. Our observations on *At*PRORP1 contrast with bacterial RNase P, where the RPR with or without its protein cofactor exhibits only a two- to ten-fold lower activity with a "pAT-type" model substrate compared to its corresponding parental pre-tRNA or even with pre-4.5S RNA [4, 21, 45]. Although these findings suggest that *At*PRORP1 might not be capable of efficiently processing substrates such as *Eco* pre-4.5S RNA [45, 46], which resembles pATSer, expression of *At*PRORP1 in an *E*. *coli* strain that is temperature sensitive (ts) for RNase P activity resulted in growth at the non-permissive temperature [9].

Third, our data suggest that N_-1_ in the substrate contributes to cleavage efficiency of and site selection by *At*PRORP1 (Table 1). Specifically, C_-1_ decreased the cleavage frequency at *C*_*0*_ both in the context of pSu1 and pATSer substrates (Fig 3). We consider two possibilities why C_-1_ might interfere with correct cleavage: (i) the exocyclic amine in C_-1_ base results in unfavorable positioning in the *At*PRORP1 active site; and (ii) formation of a C_-1_:G_+73_ bp imposes a barrier for exposing the *C*_*0*_ cleavage site, as has been suggested for bacterial RPR [14, 15, 29]. To evaluate these postulates, it is instructive to compare the frequency and rates of miscleavage of pATSerCG (C_-1_:G_+73_), pATSerCIno (C_-1_:I_+73_) and pATSerCU (C_-1_:U_+73_). With these three C_-1_ substrates, we notice a trend towards increasing correct cleavage and a higher overall rate as we transition from three to two to zero hydrogen bonds between N_-1_ and N_+73_; the four-fold higher preference for miscleavage with pATSerCG shifts to a 1.4-fold preference for correct cleavage with pATSerCU (Fig 3B; Table 1). Thus, both the identity and the strength of the bp at N_-1_:N_+73_ are important in cleavage-site selection. A few additional comments in this regard: *At*PRORP1 cleaves chloroplast pre-tRNA^Phe^ with a C_-1_:A_+73_ at *C*_*0*_ with >95% and *Tth* pre-tRNA^Gly^ with a C_-1_:U_+73_ only at *C*_*0*_ [18, 29]. In contrast, we find miscleavage (40% of total; Fig 3B) of pATSerCU; akin to the other reports, we find a bias towards correct cleavage. However, it is clear that the impact of C_-1_ appears to be dependent on context and other structural elements (for instance, shorter D and variable loops in chloroplast pre-tRNA^Phe^ and *Tth* pre-tRNA^Gly^, and a larger variable loop in *Eco* pSu1). Further support for this postulate stems from our sequence analyses (Fig 6; Table B in S1 File). While we noticed a negative bias for C_-1_:G_+73_ in that there were only 0.7% organellar tRNAs from eight different algae/plants, nearly 8% of the total suite have C_-1_. We recognize that tRNA nucleobase identities coevolve with a suite of tRNA processing and modification enzymes, including RNase P. As far as *At*PRORP1 is concerned, while C_-1_:G_+73_ is clearly not preferred, C_-1_ alone might be tolerated depending on the N_+73_ identity and other structural elements (Fig 6; Table A in S1 File; see below).

Fourth, replacement of the 2'-OH with 2'-H at N_-1_ in pATSer resulted in no detectable cleavage by *At*PRORP1 at site *C*_*0*_ (Fig C in S1 File). Because *At*PRORP1 depends on Mg^2+^ ions for activity [36–38], the 2'-OH at N_-1_ might influence positioning of functional important Mg^2+^ in the active site, as was noted earlier for bacterial RPR catalysis [31–33, 47–52]. *Eco* RPR cleaves pATSerC_deoxy_G almost exclusively at *M*_*-1*_ while pATSerU_deoxy_G is cleaved preferentially at *C*_*0*_ [31, 32; Mao and Kirsebom, unpublished data]. Hence, cleavage with *At*PRORP1 somewhat resembles the scenario with the bacterial counterpart but the identity of N_-1_ influences the magnitude of the decrease at *C*_*0*_ with bacterial RPR.

Fifth, we discovered that there is little interplay between N_-1_ and N_+1_ in cleavage-site selection by *At*PRORP1, a notable difference compared to bacterial RNase P. G_-1_-containing pSu1 and pATSer variants are cleaved with a high frequency at *M*_*-1*_ by bacterial RPR. This is particularly true for substrates having G_-1_:C_73_ (*e*.*g*. pre-tRNA^His^) [14, 15, 31, 53–57; Mao and Kirsebom, unpublished data]. G_+1_, which has been suggested to help position the nucleophile during RNase P-mediated cleavage, is indeed present in a majority of bacterial tRNAs (see *e*.*g*., http://trna.ie.niigata-u.ac.jp/cgi-bin/trnadb/index.cgi.) [34, 49, 58]. Thus, the presence of G_-1_ leads to increased cleavage at *M*_*-1*_ [15], likely due to metal-ion or other anchoring determinants now being present at both G_+1_ and G_-1_. Although organellar tRNAs from the eight green algae and plants that we analyzed also favor G_+1_ (nearly 75% bias; Table B in S1 File), it appears that *At*PRORP1 might not rely on G_+1_ as a guide for cleavage-site selection. Unlike bacterial RPR, which cleaves pSu1 and pATSer (having G_-1_ and G_+1_) at the incorrect site with either higher or similar frequencies as substrate counterparts with C_-1_ [14, 15; Mao and Kirsebom, unpublished data], *At*PRORP1 cleaved a G_-1_ substrate mainly at the correct site *C*_*0*_ [for example, see pSu1(-1G), Fig 2]. However, there is a hierarchy in cleavage-site selection by PRORPs, with contributions from multiple factors such as N_-1_ identity and the N_-1_:N_+73_ bp (especially, a G_-1_:C_+73_ bp) as evident from the following observations. In spinach chloroplast pre-tRNA^His^, G_-1_ is encoded in the gene; 5' processing of this precursor using a spinach S100 extract results in a 5'-matured tRNA^His^ with G_-1_ [59]; similarly, recombinant *At*PRORP1 cleaved potato tRNA^His^ predominantly between G_-2_ and G_-1_ [60]. This scenario with plants contrasts with yeast tRNA^His^, where G_-1_ is added after RNase P processing [61]. Also, *At*PRORP1, *At*PRORP2 and *At*PRORP3 mis-cleave (at a frequency ranging from 28% to 72%) *A*. *thaliana* nuclear pre-tRNA^Phe^ with U_-1_:A_73_ [18]. Swapping the native C_-1_:U_73_ in pre-tRNA^Gly^ to G_-1_:C_73_ led to 100% mis-cleavage at *M*_*-1*_ by *At*PRORP3 [17].

Sixth, comparing the K_D_ and k_app_ values, respectively, for binding and cleavage of pre-tRNA^Ser^Su1 and pATSerUG by *At*PRORP1 revealed the importance of the D-loop, the variable loop and the anticodon stem and loop (Fig 5 and Fig A in S1 File; Table 2). Replacement of the native T-loop (seven nt) with a GAAA tetraloop in pATSer did not affect binding but eliminated cleavage at the correct position *C*_*0*_ for both the C_-1_ and U_-1_ variants (Table 2). Hence, at least for cleavage of model hairpin-loop substrates, the T-loop equivalent contributes to the rate and fidelity but not binding. Our observations, which emphasize the importance of the T-/D-loop region for binding and processing by *At*PRORP1, are consistent with findings from earlier studies. Substitution of residues at positions 18 or 19 in the D-loop, or 56, 57 or 58 in the T-loop influenced the cleavage efficiency of *At*PRORP1 [9, 62]. A substrate in which the anticodon stem and loop is deleted was cleaved with high efficiency, whereas removal of the D-loop resulted in an RNA for which no detectable cleavage was observed [9]. Footprinting analysis of pre-tRNA^Cys^ further indicated that U16, G18, G19 (D-loop) and C56 (T-loop) are protected when bound to *At*PRORP1 [11]. Moreover, the K_M(STO)_ and k_react_ (kinetic constants determined under single turn over) of *At*PRORP3-mediated processing of pre-tRNA^Gly^ decreased by 1200- and 26-fold, respectively, upon deletion of the D-stem-loop and anticodon stem-loop [17]. Taken together, it is clear that efficient and correct cleavage depends on a productive interaction between the T-/D-loop region and *At*PRORP1, as has been shown for bacterial RPR [7, 21, 22; see also 51, 63–65].

Last, we find that the absence or presence of 3'-CCA in either pSu1 or pATSer does not affect cleavage-site selection by *At*PRORP1 (Fig 3). The rate of cleavage, however, does change by two- to three-fold but not in any predictable fashion with the various substrates that we studied (Table 1). *At*PRORP1-mediated cleavage of a plant mitochondrial pre-tRNA^Cys^ was shown to be inhibited by the presence of a 3'-RCCA motif [11]. Together, these results with *At*PRORP1 emphasize an important difference compared to bacterial RNase P, where the rate and fidelity of cleavage are dramatically affected when the 3'-CCA is deleted from either a pre-tRNA or "pAT-type" substrate; these results are expected due to the base pairing between the 3'-RCC of the pre-tRNA and a conserved GGU-motif in the RPR [13, 66]. Unlike bacterial pre-tRNAs, a 3'-CCA was predicted to be present in the initial pre-tRNA transcript for only 0.5% of the total suite of 423 organellar genome-encoded pre-tRNAs in eight plant/algal species. Thus, the 3'-CCA is unlikely to be a major contributor to *At*PRORP1 catalysis (see also [18]).

### Substrate recognition by the bacterial ribozyme variant and At*PRORP1*

For bacterial RNase P, biochemical and genetic studies have provided insight into substrate recognition features, and these were confirmed and extended by the crystal structure of the bacterial RNase P-tRNA complex [51]: (i) N_-1_ in the pre-tRNA has a key role and might interact with a specific base in the RPR; (ii) the 2'-hydroxyl N_-1_ is used to coordinate metal ions essential for catalysis; (iii) G_+1_ in the pre-tRNA acts as a guidepost in the RNase P-substrate complex; (iv) the T-loop in the pre-tRNA is specifically recognized by an architectural motif of two inter-digitated T-loops in the RPR; and (v) 3'-RCC sequence of the pre-tRNA pairs with a conserved GGU sequence in the RPR [51]; for reviews see *e*.*g*. [1, 11]. The anticodon stem-loop was shown to be dispensable, which is expected given that all tRNAs are processed by RNase P. Two previous models [11, 42] show how *At*PRORP1 might use the "acceptor-T-stem" stack as the main recognition determinant, an idea that is supported by our finding here that the pATSer-type variants, which possess only the acceptor-T-stem stack element, are cleaved by *At*PRORP1 with the same fidelity as the parental tRNA counterparts (Fig 3 and Table 3).

**Table 3:** Comparison of substrate recognition attributes of bacterial RNase P (RNP) and PRORPs.

Pre-tRNA location	Role in catalysis	Bacterial RNase P	PRORP	References
5'-leader	Substrate recognition	From N_-1_ to N_-7_	Only N_-1_ and N_-2_	[17, 18, 51]
N_-1_ identity	Cleavage fidelity and efficiency	Yes		This study and [7, 15, 17]
2'-OH in N_-1_	Cleavage efficiency	Yes		This study and [31, 32]
G_+1_ as positive and G_-1_ as negative determinants	Cleavage-site selection	Yes	No	This study and [14, 15, 29]
N_-1_:N_73_ base pairing	Cleavage fidelity	Yes		This study and [14, 15, 17, 18, 29]
D-stem/loop	Rate of cleavage	Moderate	Significant	This study and [4, 11, 17, 18, 21]
T-stem/loop	Rate of cleavage	Significant		This study and [4, 11, 17, 21]
3'-CCA motif	Substrate recognition and cleavage fidelity	Yes	No	This study and [9, 13, 17, 37]

With respect to pre-tRNAs, however, *At*PRORP1 is likely to interact with the amino acceptor-stem and the T-/D-loop region (Table 3). It is possible that the distance between T-/D-loop region and cleavage site determines metal-ion binding and cleavage-site selection by *At*PRORP1 [11], but this notion might need refinement if *At*PRORP1 (like the bacterial RPR) accepts substrates with shorter acceptor-/T-stem stacks. Additional insights are needed to ascertain whether this amounts to a measuring mechanism that has been suggested for bacterial and eukaryotic RNase P [58, 63, 67, 68]. Since we observe binding of both pATSerUG and pATSerUG_GAAA_ but cleavage of only the former (Table 2; Fig 5), an induced-fit mechanism based on T-loop recognition is likely with *At*PRORP1, again mirroring a proposal for bacterial RNase P [7, 21, 22].

Bacterial RPR/RNase P uses multiple determinants to define its cleavage site whereas *At*PRORP1 appears to employ fewer elements and differs notably in not using either the 5'-leader or the 3'-trailer (Table 3) [17, 18]. While this difference might signify how binding energy and cleavage-site selection are accomplished by nucleic acid- versus protein-based RNase P, it likely reflects the culmination of a catalytic strategy based on the co-evolution of each catalyst with its entire suite of substrates not just pre-tRNAs. Both forms of RNase P have honed in on the common denominators in all pre-tRNAs: the acceptor-T-stem stack and the T-/D-loop interaction [69], which incidentally is used as a recognition determinant by other RNAs and proteins that act on tRNA [70].

## Supporting Information

S1 File Tables A and B, Figs A-C with figure legends. (PDF)
