# Supplementary material for: Cleavage of Model Substrates by Arabidopsis thaliana PRORP1 Reveals New Insights into Its Substrate Requirements
Source: PLoS One. 2016 Aug 5;11(8):e0160246. doi: 10.1371/journal.pone.0160246 (PMC4975455; doi:10.1371/journal.pone.0160246)
Supplement: S1 File — (PDF) [file pone.0160246.s001.pdf]

# Supporting information

# Cleavage of model substrates by *Arabidopsis* PRORP1 reveals new insights into its substrate requirements

Guanzhong Mao[a,*], Tien-Hao Chen[b,*], Abhishek S. Srivastava[a,c], David Kosek[a], Pradip K. Biswas[b], Venkat Gopalan[b] and Leif A. Kirsebom[a,**]

**Supporting Tables A-B and Figures A-C**

**Table A**

Analysis of $N_{-1}$:$N_{+73}$ identities in mitochondrial and chloroplast tRNAs from eight different green algae and plants

Composite (total of 423 tRNAs)

| $N_{-1}$ \ $N_{+73}$ | A | U | G | C |
|---|---|---|---|---|
| **A** | 78 | 27 | 42 | 9 |
| **U** | 77 | 32 | 40 | 11 |
| **G** | 42 | 10 | 15 | 8 |
| **C** | 23 | 5 | **3** | 1 |

*Arabidopsis thaliana*

| $N_{-1}$ \ $N_{+73}$ | A | U | G | C |
|---|---|---|---|---|
| **A** | 7 | 3 | 5 | 4 |
| **U** | 5 | 2 | 5 | 1 |
| **G** | 9 | 2 | 1 | 2 |
| **C** | 2 | 1 | **1** | 0 |

*Cyanophora paradoxa*

| $N_{-1}$ \ $N_{+73}$ | A | U | G | C |
|---|---|---|---|---|
| **A** | 12 | 5 | 8 | 0 |
| **U** | 15 | 5 | 8 | 3 |
| **G** | 1 | 1 | 1 | 0 |
| **C** | 2 | 1 | **0** | 1 |

*Ectocarpus siliculosus*

| $N_{-1}$ \ $N_{+73}$ | A | U | G | C |
|---|---|---|---|---|
| **A** | 10 | 5 | 4 | 2 |
| **U** | 12 | 7 | 4 | 1 |
| **G** | 3 | 0 | 3 | 0 |
| **C** | 1 | 0 | **0** | 0 |

*Oryza sativa japonica*

| $N_{-1}$ \ $N_{+73}$ | A | U | G | C |
|---|---|---|---|---|
| **A** | 9 | 4 | 5 | 1 |
| **U** | 8 | 3 | 4 | 0 |
| **G** | 6 | 0 | 5 | 3 |
| **C** | 2 | 0 | **0** | 0 |

*Ostreococcus tauri*

| $N_{-1}$ \ $N_{+73}$ | A | U | G | C |
|---|---|---|---|---|
| **A** | 2 | 2 | 8 | 0 |
| **U** | 13 | 5 | 5 | 3 |
| **G** | 2 | 0 | 1 | 0 |
| **C** | 8 | 0 | **2** | 0 |

*Phaeodactylum tricornutum*

| $N_{-1}$ \ $N_{+73}$ | **A** | **U** | **G** | **C** |
|---|---|---|---|---|
| **A** | 14 | 2 | 4 | 1 |
| **U** | 10 | 5 | 3 | 1 |
| **G** | 2 | 1 | 1 | 0 |
| **C** | 5 | 1 | **0** | 0 |

*Physcomitrella patens*

| $N_{-1}$ \ $N_{+73}$ | **A** | **U** | **G** | **C** |
|---|---|---|---|---|
| **A** | 15 | 4 | 4 | 1 |
| **U** | 9 | 3 | 5 | 1 |
| **G** | 8 | 2 | 0 | 1 |
| **C** | 1 | 1 | **0** | 0 |

*Solanum tuberosum*

| $N_{-1}$ \ $N_{+73}$ | **A** | **U** | **G** | **C** |
|---|---|---|---|---|
| **A** | 9 | 2 | 4 | 0 |
| **U** | 5 | 2 | 6 | 1 |
| **G** | 11 | 4 | 3 | 2 |
| **C** | 2 | 1 | **0** | 0 |

For the sequence analysis above (Tables S1 and S2), we first obtained tRNA sequences from http://plantrna.ibmp.cnrs.fr/. We examined eight algal/plant species that appear to have the entire suite of organellar tRNAs. We filtered duplicated genes, classified as such by the presence of identical upstream and body sequences. All the tRNA sequences were downloaded, collated using a Microsoft Excel spreadsheet, and analyzed for identity distributions at desired locations.

**Table B**

Analysis of $N_{+1}$ identities of mitochondrial and chloroplast tRNAs from eight different green algae and plants

| | **A** | **U** | **G** | **C** |
|---|---|---|---|---|
| *Ath* (50) | 10 | 4 | 34 | 2 |
| *Cpa* (63) | 9 | 5 | 44 | 5 |
| *Esi* (52) | 0 | 6 | 43 | 3 |
| *Osa* (50) | 7 | 4 | 38 | 1 |
| *Ota* (51) | 3 | 5 | 41 | 2 |
| *Ptr* (50) | 8 | 4 | 35 | 3 |
| *Ppa* (55) | 6 | 3 | 43 | 3 |
| *Stu* (52) | 7 | 5 | 38 | 2 |
| **Total** (423) | **50** | **36** | **316** | **21** |

Abbreviations used: *Ath, Arabidopsis thaliana*; *Cpa, Cyanaphora paradoxa*; *Esi, Ectocarpus siliculosus*; *Osa, Oryza sativa*; *Ota, Ostreococcus tauri*; *Ptr, Phaeodactylum tricornutum*; *Ppa, Physcomitrella patens; Stu, Solanum tuberosum*.

**Suppportin figure legends**

*Figure A*

Representative gels showing *At*PRORP1-mediated cleavage of pATSer variants, lacking the 3' CCA-motif. Lanes 1 to 2 and 6 to 9 are negative controls (loaded in the same order as with *At*PRORP1); M (size marker lanes 3 and 10) indicates cleavage of pATSerUG by *Eco* RPR. Lane 4, pATSerCG$_{\Delta 3'CCAC}$; lane 5, pATSerCIno$_{\Delta 3'CCAC}$; lane 11, pATSerUG$_{\Delta 3'CCAC}$; lane 12, pATSerUG$_{\Delta 3'CAC}$; lane 13, pATSerUG$_{\Delta 3'AC}$; and lane 14, pATSerUG. The final concentration of *At*PRORP1 was 6.6 μM and the reactions were performed in the presence of 10 mM $Mg^{2+}$ for 60 min at 37°C (for details, see Materials and Methods). The position of each 5' cleavage fragment (5' CL Frags) is indicated.

*Figure B*

Representative data from fluorescence polarization binding assays performed with *At*PRORP1 and 3'-fluorescein-labeled pSu1(-1U), pATSerUG and pATSerUG$_{GAAA}$.

*Figure C*

Representative gels showing *At*PRORP1-mediated cleavage of pATSerUG and pATSerCG with 2'OH and 2'H at $N_{-1}$. Lanes 1 to 4 are negative controls (loaded in the same order as with *At*PRORP1); and M (size marker, lane 5) indicates cleavage of pATSerUG by *Eco* RPR. Lane 6, pATSerUG; lane 7, pATSerCG; lane 8, pATSerU$_{deoxy}$G; and lane 9, pATSerC$_{deoxy}$G. The final concentration of *At*PRORP1 was 11 μM and the reactions were performed in the presence of 10 mM $Mg^{2+}$ for 60 min at 37°C (for details, see *Materials and Methods*). The position of each 5' cleavage fragment (5' CL Frags) is indicated.

# Fig A

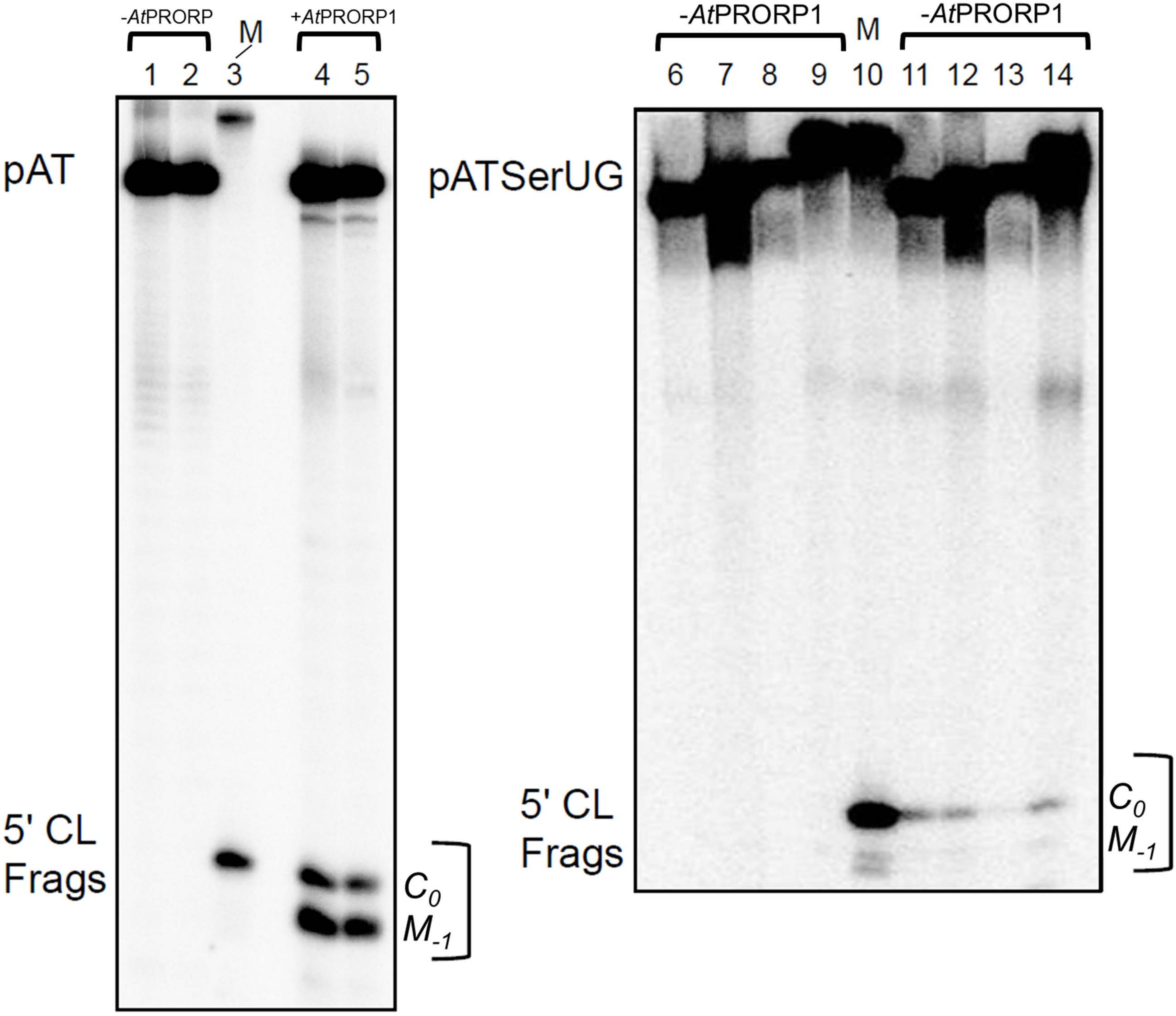

-AtPRORP
M
+AtPRORP1
1
2
3
4
5
pAT
5' CL
Frags
$C_0$
$M_{-1}$
-AtPRORP1
M
-AtPRORP1
6
7
8
9
10
11
12
13
14
pATSerUG
5' CL
Frags
$C_0$
$M_{-1}$

# Fig B

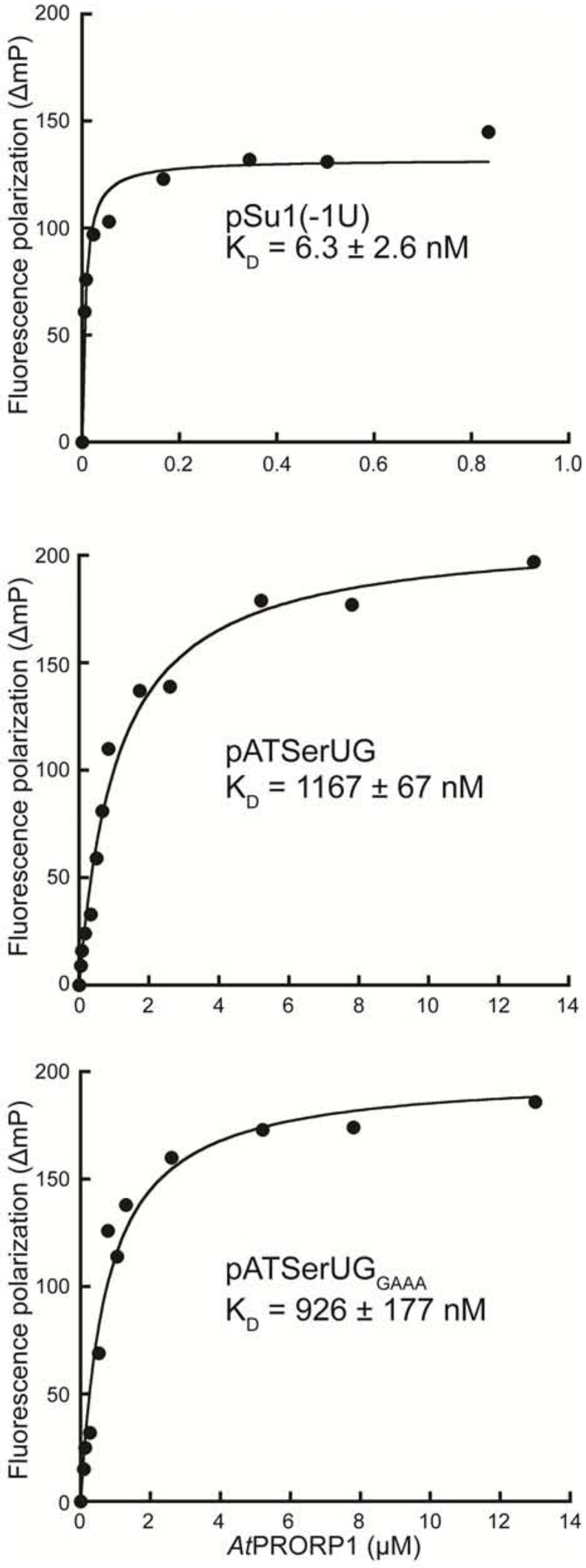

Fluorescence polarization (ΔmP)
pSu1(-1U)
$K_D$ = 6.3 ± 2.6 nM
pATSerUG
$K_D$ = 1167 ± 67 nM
pATSerUG$_{GAAA}$
$K_D$ = 926 ± 177 nM
*At*PRORP1 (μM)

Fig C

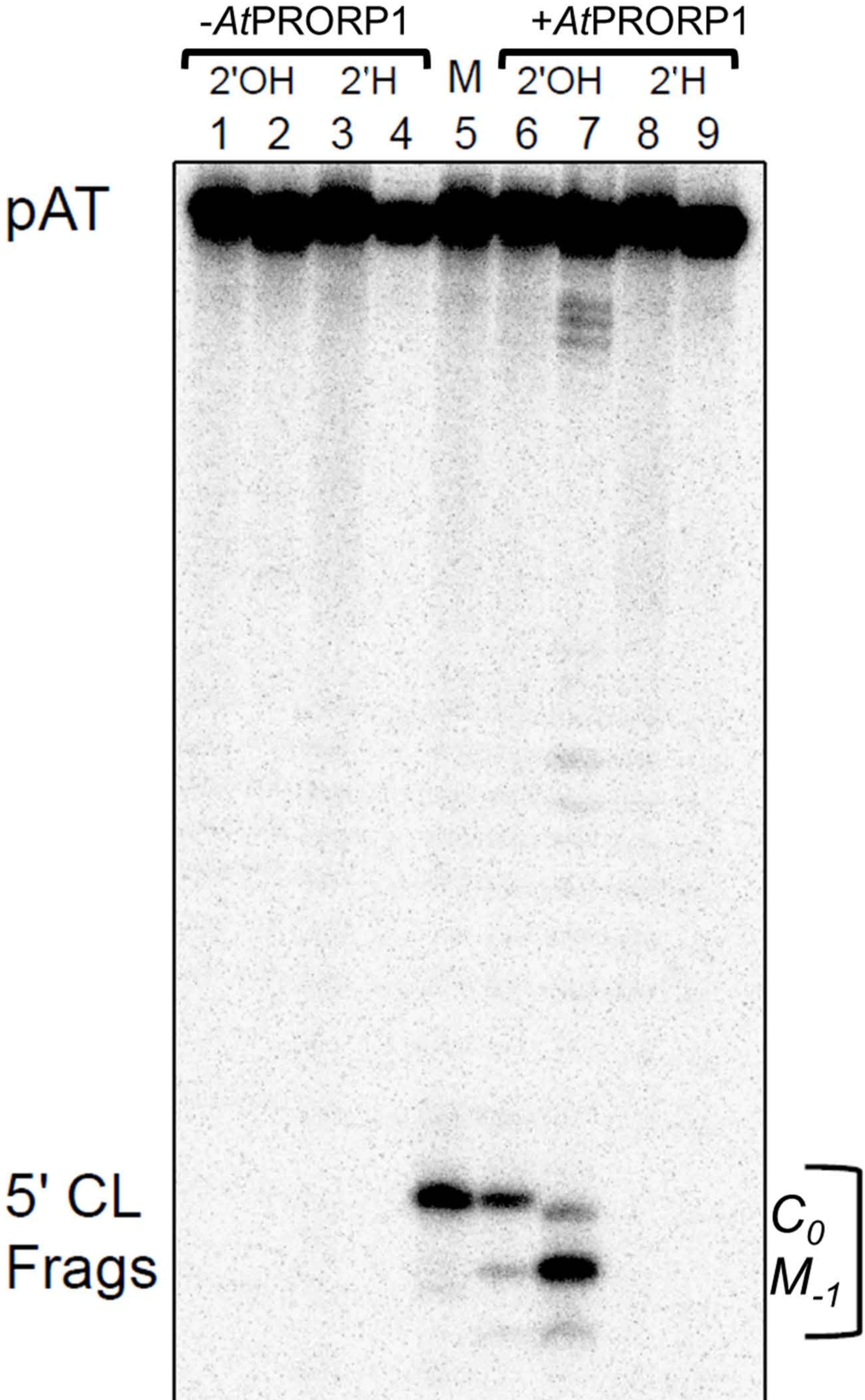
-*At*PRORP1
+*At*PRORP1
2'OH
2'H
M
2'OH
2'H
1
2
3
4
5
6
7
8
9
pAT
5' CL
Frags
$C_0$
$M_{-1}$
